# An intervention to improve program implementation: findings from a two-year cluster randomized trial of Assets-Getting To Outcomes

**DOI:** 10.1186/1748-5908-8-87

**Published:** 2013-08-07

**Authors:** Joie Acosta, Matthew Chinman, Patricia Ebener, Patrick S Malone, Susan Paddock, Andrea Phillips, Peter Scales, Mary Ellen Slaughter

**Affiliations:** 1 RAND Corporation, 4570 Fifth Avenue, Pittsburgh PA 15213, USA; 2 University of South Carolina, 920 Sumter St, Columbia SC 29208, USA; 3 Search Institute, 615 First Avenue NE, Suite 125, Minneapolis MN 55413, USA

**Keywords:** Implementation support, Technical assistance, Capacity building

## Abstract

**Background:**

Studies have shown that communities have not always been able to implement evidence-based prevention programs with quality and achieve outcomes demonstrated by prevention science. Implementation support interventions are needed to bridge this gap between science and practice. The purpose of this article is to present two-year outcomes from an evaluation of the Assets Getting To Outcomes (AGTO) intervention in 12 Maine communities engaged in promoting Developmental Assets, a positive youth development approach to prevention. AGTO is an implementation support intervention that consists of: a manual of text and tools; face-to-face training, and onsite technical assistance, focused on activities shown to be associated with obtaining positive results across any prevention program.

**Methods:**

This study uses a nested and cross-sectional, cluster randomized controlled design. Participants were coalition members and program staff from 12 communities in Maine. Each coalition nominated up to five prevention programs to participate. At random, six coalitions and their respective 30 programs received the two-year AGTO intervention and the other six maintained routine operations. The study assessed prevention practitioner capacity (efficacy and behaviors), practitioner exposure to and use of AGTO, practitioner perceptions of AGTO, and prevention program performance. Capacity of coalition members and performance of their programs were compared between the two groups across the baseline, one-, and two-year time points.

**Results:**

We found no significant differences between AGTO and control group’s prevention capacity. However, within the AGTO group, significant differences were found between those with greater exposure to and use of AGTO. Programs that received the highest number of technical assistance hours showed the most program improvement.

**Conclusions:**

This study is the first of its kind to show that use of an implementation support intervention-AGTO -yielded improvements in practitioner capacity and consequently in program performance on a large sample of practitioners and programs using a randomized controlled design.

**ClinicalTrials.gov identifier:**

NCT00780338

## Background

Prevention programming can improve youth outcomes and be cost effective [1]. While programs need to be well implemented, they also require practices such as tracking outcomes and engaging in continuous improvement-to reap these benefits [2]. Yet, many studies have shown that communities have not been able to implement evidence-based prevention programs with such quality and achieve outcomes demonstrated by prevention science [3-8]. This *‘*gap*’* between science and practice (e.g., [9,10]) can result when communities lack individuals with the capacity-defined as the self-efficacy and behaviors-needed to engage in critical prevention practices.

**Figure 1:**
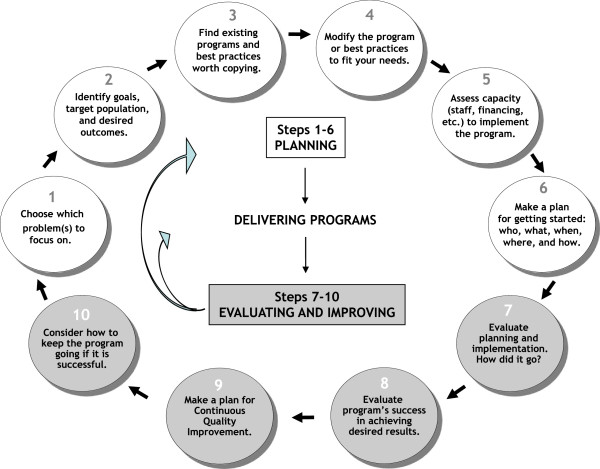
GTO 10 steps.

Getting To Outcomes® (GTO®)^a^ was developed to bridge this gap between science and practice. GTO is an implementation model (Figure 1)-specifying ten steps (or sets of activities) prevention practitioners should take, shown to be associated with obtaining positive results across many different prevention programs [11]. The first six involve planning activities (needs assessment, goal setting, choosing programs, ensuring appropriate capacity and fit, planning program details), the next two steps are process and outcome evaluation [7,8], and the last two steps involve using data to improve programs and sustaining programs [9,10]. GTO is also an implementation support intervention, which strengthens the efficacy and behaviors (collectively known as capacity) community practitioners need to perform these activities with quality. GTO has been found to improve the capacity of individual practitioners and the performance of drug prevention programs in both quasi-experimental [12] and randomized controlled trials [13]. Congruent with social cognitive theories of behavioral change [14-17], the efficacy and behaviors related to the activities targeted by GTO’s 10 steps (i.e., prevention *‘*capacity*’* at the individual level), are related to how well prevention is carried out at the program level [18], which, in turn, affects outcomes.

GTO has been adapted to a number of content areas, recently to the model of positive youth development developed by Search Institute. This model, called *‘*Developmental Assets*,’* states that in order to thrive, young people need 40 key developmental supports and experiences that include but are broader than the notion of protective factors. From a caring school climate to participation in high-quality after-school programs, these factors have been shown to predict health and well-being outcomes consistently across sex, race-ethnicity, and family income [19-21]. This approach was blended into the 10 GTO steps, so that each step was enhanced to include a specific focus on building assets. For example, in step one (choose which problem(s) to focus on) individuals use both asset indicators, as well as risk information, to help select priorities. This blended approach is called Assets-Getting To Outcomes (AGTO) [22].

The AGTO model and corresponding intervention is grounded in implementation theory. For example, AGTO operationalizes the Consolidated Framework for Implementation Research (CFIR) to ensure that all the major domains that influence implementation are considered [23]. Intervention characteristics (e.g., evidence strength and quality, relative advantage, complexity, adaptability), the first domain, can influence whether practitioners adopt an intervention. Without adaptation, many interventions come to settings as a poor fit. Even if these interventions have been known to improve outcomes, practitioners do not always adopt these interventions, underscoring the need for core intervention elements and what the CFIR terms an adaptable intervention periphery (i.e., elements that can be adapted to improve intervention fit [3]. The AGTO intervention walks practitioners through a systematic process to assess, and then improve, how well interventions fit with their target population, organization, and broader community (step four: Fit).

The next three domains of the CFIR comprise the outer setting (e.g., broader social, political and economic context including policies, incentives, resources etc.), inner setting in which the intervention is implemented (e.g., context of the specific organization or group implementing the intervention including the structural characteristics, relationships, implementation readiness), and the characteristics of the individuals involved (e.g., knowledge, skills). Attention needs to be paid to factors both at the individual and organizational levels that impact the degree to which new practices are adopted and implemented. For example, a number of studies have shown that factors at both the individual level (e.g., training, skills, efficacy, involvement in decision making, and satisfaction of teachers) and at the organizational level (organization size, climate, and financial resources, and active support of principals and administrators) have been shown to predict successful implementation of prevention in public schools [3,24-28]. In the AGTO study, the inner setting is the coalition, the outer setting is the broader community where coalitions are located, and the individuals involved are the prevention practitioners who are coalition members. To create both capacity and conditions for successful implementation of programs, the AGTO intervention targets both practitioners and coalitions. While AGTO helps the inner setting and individuals account for the outer setting, it is beyond its scope to specifically alter the broader social, political, and economic context.

Implementation process or the active change process aimed to achieve individual and organization use of the intervention as designed is the final domain in the CFIR. Research suggests that innovative multi-level strategies are needed to improve the implementation process. Passive approaches such as trainings by themselves do not lead to change, as attendees often experience barriers to incorporating newly learned information into their work [29,30]. The AGTO intervention, which is proactive versus passive, specifically targets improvements to this implementation process at both the individual and program level so that implementation more closely aligns with empirically-based, high-quality prevention processes.

The purpose of this article is to present two-year outcomes from an evaluation of AGTO in 12 Maine communities engaged in promoting Developmental Assets (interim one year outcomes have been presented elsewhere [18]). At random, six coalitions and their respective programs received the two-year AGTO intervention and the other six maintained routine operations. Capacity of coalition members and performance of their programs were compared between the two groups across the baseline, one-, and two-year timepoints. The study had the following hypotheses:

Hypothesis one: Individual prevention practitioners in the AGTO group would demonstrate greater gains in capacity than those in the control group.

Hypothesis two: Individual prevention practitioners with greater exposure to and use of AGTO would demonstrate greater gains in capacity than those who were assigned to use AGTO but used less or none.

Hypothesis three: Whole prevention programs in the AGTO group would demonstrate significant gains in prevention performance (i.e., performing various prevention tasks with high quality) overall and in proportion with their use of AGTO.

Building upon previous GTO studies, the current study is the first to evaluate GTO on both individual capacity and program performance in a randomized trial with a larger number of programs in a way that uses a traditional approach to conducting a randomized trial, while being flexible enough to account for the *‘*complex, interacting, multi-level, and transients states of constructs in the real world*’*[31]. Consistent with CFIR, the study attempts to evaluate AGTO in terms of its impact on three of the CFIR domains: inner setting, individual characteristics, and implementation process; while using measures of the outer setting as covariates. Table 1 provides a summary of how the study design aligns with and measures the CFIR domains.

**Table 1:** CFIR domains by study design and measures

**CFIR domains**	**How the domain aligns with AGTO**	**Relevant study measures**
Intervention characteristics	AGTO walks practitioners through a systematic process to assess, and then improve, how well interventions fit with their target population, organization, and broader community	No study-wide measures, but AGTO supported the development of process measures to assess these characteristics at the program level
Outer setting	Broader social, economic, and political context of the 12 participating communities, outside the scope of AGTO	Practitioners perceptions of how community context affected program implementation (qualitative interviews)
Individuals involved	AGTO builds the prevention capacity of coalition members and program staff that comprise the study sample	Practitioners prevention self-efficacy with AGTO and AGTO behaviors (coalition survey)
Inner setting	AGTO targets improving the setting of interventions within the 12 community-based coalitions and 60 programs that comprise the study sample	Practitioners satisfaction with coalition membership and leadership (coalition survey)
Implementation process	AGTO proactively targets improvements to the implementation process at both the individual and program level so that implementation more closely aligns with empirically-based high quality prevention processes	Self-reported exposure to AGTO (coalition survey) and utilization of AGTO TA (TA providers log) Program implementation along the 10 steps of AGTO (capacity interview)

## Methods

### Study design

The study was a nested cross-sectional matched pairs group randomized trial [32]. Twelve coalitions were randomized to either receive the two-year AGTO intervention or to serve as comparison coalitions. Prior to randomization, coalitions were matched into pairs based on the total population and demographic characteristics of the community served by each coalition (from the 2000 Census) and a rating of each coalition’s functioning at baseline. Two technical assistance providers and a local coalition expert came to consensus to derive the pairings based on each participating coalition’s overall stage of development, as well as their stage of development in four functional areas: coalition development and management, coordination of prevention programs and services, conducting environmental strategies, and acting as intermediary or support organization to other community organizations and coalitions. Coalitions were rated on their mastery of each functional area on a Likert scale. The matched pairs were sent to the project statistician, who allocated one coalition per matched pair to the AGTO intervention by using the random number generator in Microsoft Excel. Programs and practitioners followed their coalition assignment. The rating and resulting four-stage categorization were developed by Office of National Drug Control Policy’s evaluation of the Drug-Free Communities program after reviewing the literature on coalition functioning and receiving input from experts and coalition leaders [33]. Analysis of these stages within the Drug-Free Communities program showed that the more advanced coalitions had stronger prevention practices and better outcomes [33]. As will be seen below, it was not possible to blind research staff or participants to their study condition.

### Setting: 12 community-based coalitions

Participants were all the coalition members and program staff from 12 coalitions in Maine. Community-based coalitions are comprised of prevention practitioners from non-profit organizations from multiple levels (individual, organizational, policy) and sectors (parents, youth, criminal justice, education) that collaborate to improve community health [34]. These 12 coalitions are similarly structured (a small group of paid staff working with a larger group of volunteers), work in similar geographic and demographics settings,^b^ and have similar rates of reported youth risk behaviors (e.g., alcohol and drug use),^c^ missions (to promote healthy youth development), annual budgets, and numbers of distinct programs. However, it is important to note that membership and leadership in coalitions are not static, but change over time. These coalitions represent the inner setting defined by the CFIR.

### Participants

#### Prevention practitioners

Coalition members, characterized by the CFIR as the individuals involved, completed a coalition survey at baseline (just prior to the AGTO intervention, April 2009), mid (about one year later, June 2010) and post (about two years later, July 2011). After randomization, but prior to completing the survey, we received approval from RAND’s Human Subjects Protection Committee for the study and written consent was obtained from all participants. The overall response rate was 82% across all sites at baseline, 79% at mid, and 89% at post. Community coalitions often experience high turnover and the same was true here, as 41% percent of those surveyed at baseline or mid were also surveyed at post. New participants joined the coalition after baseline as well. There were 376 survey participants at baseline, 303 at mid, and 315 at post (152 of whom had participated in the baseline survey and 163 who had not), yielding a total of 591 unique participants. The size of the clusters used in the analyses (in this case, coalitions) ranged from n = 8 to n = 50 across the 12 coalitions and three time points. In lieu of a CONSORT diagram (Additional file 1), Table 2 presents additional response rate and sample size information is, which better presents this information for this study. At baseline and post, we compared the distributions of several participant characteristics (see covariates below) between the intervention and control groups using ordinal, logistic, or linear regression, with the AGTO group indicator as a predictor variable (see Table 3). We found no statistically significant differences in characteristics between the groups at either baseline or post, except for satisfaction with involvement in the coalition. The AGTO group reported significantly higher satisfaction at post than the comparison group (p = 0.003). Analyses were adjusted to account for this statistically significant difference, and given that it was one of a large number of t-tests we do not anticipate this difference influenced the findings presented in this article.

**Table 2:** Additional sample size information

		**Response rates, %**			**Use of AGTO, %**		**Sample sizes**						
**Coalition pairs**	**Study condition**	**Baseline**	**Mid**	**Post**	**First year of AGTO**	**Second year of AGTO**	**Baseline**	**Mid**	**Post**	**All time-points**	**Baseline and mid**	**Mid and post**	**Baseline and post**
1a	Control	72.09	57.69	90.00	10	29	31	14	27	27	6		2
1b	AGTO	93.33	100.00	88.89	93	85	14	20	8	12	18	2	
2a	Control	70.83	61.90	81.25	18	23	17	13	13	18	10		
2b	AGTO	81.82	86.67	93.55	47	50	17	26	29	39	6	10	
3a	Control	80.65	81.40	92.86	12	17	50	35	39	54	14	8	2
3b	AGTO	83.87	80.39	84.62	34	49	50	41	33	60	14	12	
4a	Control	95.24	78.05	83.33	23	32	40	31	30	45	16	14	
4b	AGTO	80.00	72.73	88.89	21	47	24	15	24	27	2		
5a	Control	74.36	74.36	82.98	17	24	29	29	39	42	10	12	
5b	AGTO	87.18	85.00	95.12	32	38	34	34	39	60	4	14	
6a	Control	79.55	70.59	100.00	9	25	35	12	11	12	12		
6b	AGTO	87.80	89.74	88.46	20	24	35	33	23	54	12	6	

**Table 3:** **Characteristics of study sample, AGTO versus non-AGTO**^**h**^

	**Baseline**		**Post**	
	**AGTO (n = 174)**	**Non-AGTO (n = 202)**	**AGTO (n = 159)**	**Non-AGTO (n = 156)**
Age in years, %				
12-49	53	53	46	48
50+	47	47	55	52
Female, %	72	74	71	74
White, %	98	97	97	100
Education, %				
HS graduate or less	6	12	4	6
Some college	22	6	21	10
College graduate	21	27	27	32
Graduate education	50	54	49	51
Employment status, %				
Full time	81	78	78	81
Part time	13	9	13	12
Out of labor force	6	12	10	7
Coalition leadership (0–100), mean (SD)	77.4 (16.1)	71.9 (19.1)	80 (17.7)	74 (18.8)
Coalition cohesion (0–100), mean (SD)	76.0 (15.4)	71.9 (14.3)	77 (13.4)	72 (14.8)
Coalition receptivity to change (0–100), mean (SD)	72.9 (13.0)	69.1 (13.7)	74 (11.5)	69 (13.4)
Satisfaction with involvement in coalition, %				
1 = Very dissatisfied, 2, 3	6	9	2	6
4	9	16	7	19
5	17	16	13	19
6	41	43	44	46
7 = Very satisfied	27	17	34	11
Years in coalition, %				
0	7	13	9	21
1	25	25	8	2
2	16	16	14	20
3	7	10	15	13
4	5	13	9	11
5	8	6	8	10
6+	32	18	36	24
Type of involvement, %				
Paid staff	31	23	26	24
Volunteer individual	21	33	32	34
Volunteer from partner organization	49	45	42	42

^h^There were no statistically significant differences between AGTO and non-AGTO study participants on almost all of these characteristics at baseline or post (p>0.05), except for satisfaction with involvement in coalition.

### Prevention programs

Each coalition nominated up to five prevention programs to participate in the study (one-half of the programs were assigned to intervention group, one-half to the control group). Despite some variability, all programs aimed to promote healthy development with middle and high school youth. Although some were evidence-based programs including Project ALERT [35], Lifeskills Training [36], and Making Proud Choices [37-39], most were designed by coalitions, including mentoring, social norms campaigns, programs to divert first offenders from the juvenile justice system, and leadership training. Most programs were ongoing at the outset of the study.

### AGTO intervention

The AGTO intervention includes three types of assistance which are adapted to fit the needs and priorities of the individuals involved, as well as the inner and outer setting: a manual of text and tools; face-to-face training, and onsite technical assistance (TA). These three types of assistance aim to improve the implementation process for each program. Two full-time, Maine-based staff, one with a master’s and one with a bachelor’s degree, provided AGTO tools, training, and TA to the intervention coalitions and programs during the two-year intervention period. The tools are in the Search Institute-published manual, *Getting To Outcomes with Developmental Assets: Ten steps to measuring success in youth programs and communities*[22], which all intervention participants received. The training was delivered separately to each coalition over a full day after baseline, and covered the AGTO model, tools in the manual, and an introduction to the TA process. Based on TA literature [40-43], the AGTO-based TA involves three structured steps, including an initial diagnosis of program functioning, development of a logic model, and development of a plan for how the TA and program staff were to make improvements, carried out during and in between bi-weekly TA visits. TA staff provided consultation and feedback to practitioners on conducting AGTO tasks as applied to their program [44-46]. An expanded discussion about the AGTO intervention is available elsewhere [18].

### Measures

#### Covariates

The coalition survey included demographic information about prevention practitioners (i.e.*,* age, gender, race/ethnicity, education, and employment status), consistent with CFIR’s individual characteristics domain. Measures related to coalition functioning, leadership, and the incorporation of new practices were also included in the coalition survey, and relates to the inner setting domain of CFIR. The leadership score [47] is the mean of ten items rating the effectiveness of coalition leadership from five response options (1 = *‘*poor*’* to 5 = *‘*excellent*’*), α_BL_ = 0.96, α_mid_ = 0.95, α_post_ = 0.96. From the staff survey of organizational readiness for change measure [48], *Cohesion* (α_BL_ = 0.86, α_mid_ = 0.82, α_post_ = 0.86) and receptivity to change scores (α_BL_ = 0.73, α_mid_ = 0.72, α_post_ = 0.75) were adapted, averaging across five response options (1 = *‘*strongly disagree*’* to 5 = *‘*strongly agree*’*). Stand-alone coalition survey items used in the analysis were overall satisfaction with involvement in the coalition (1 = ‘very dissatisfied’ to 7 = ‘very satisfied’), years of involvement in the coalition, and type of involvement (paid staff, volunteer individual, or volunteer from a partner organization).

### Practitioner prevention capacity

Assessed in the coalition survey, prevention capacity was defined as efficacy and behaviors of practitioners and relates to CFIR’s individual characteristics domain. There are two scales that assess perceived efficacy (i.e., completing various tasks without assistance) using a three-point scale (1 = *‘*would need a great deal of help to carry out this task*,’* 2 = *‘*could carry out this task, but would need some help*,’* 3 = *‘*could carry out this task without any help*’*). The asset efficacy scale includes five items asking about helping adults connect with youth; involving multiple sectors of the community; engaging youth in asset-building; incorporating assets into existing programs; and helping community leaders understand how their decisions affect the development of youth (α_BL_ = 0.81, α_mid_ = 0.86, α_post_ = 0.82). The GTO efficacy scale includes items asking about activities from the GTO steps -e.g., evaluation, planning, quality improvement (α_BL_ = 0.84, α_mid_ = 0.89, α_post_ = 0.88).

There are three behavior scales that are means of items with seven-point scales (1 = *‘*never*’* to 7 = *‘*very often*’*) assessing the frequency with which respondents engaged in these activities during the previous 12 months. The Asset Behaviors scale includes five items assessing whether individuals are motivating both adults and youth to become asset-builders; incorporating asset building into existing youth programs; influencing community leaders to implement asset-aligned policies (e.g., employ funding criteria that rewards asset-building); and engaging various sectors of the community to support asset building (α_BL_ = 0.91, α_mid_ = 0.93, α_post_ = 0.93). GTO behaviors include 11 items that align with the 10 steps (two items focus on needs and resources assessment) (α_BL_ = 0.92, α_mid_ = 0.94, α_post_ = 0.95). AGTO behaviors includes 11 items that tailor the GTO 10 steps to Assets (e.g., needs assessment on youth assets) (α_BL_ = 0.94, α_mid_ = 0.96, α_post_ = 0.95). The GTO scales are shortened versions of scales used in previous studies (e.g., [12]). All Assets scales were developed for the current study, based on the structure used for the GTO scales. Although the goal of the intervention was build practitioners’ capacity related to the blended AGTO model, GTO and assets efficacy and behaviors were asked about separately to identify which aspects of their capacity improved and to avoid masking potential effects of the intervention (e.g., practitioners that apply GTO to other areas of their work life, may have disproportionately greater increases in capacity related to GTO).

### Use of and exposure to AGTO

To assess AGTO’s potential impact on each program’s implementation process, we first documented the degree to which each program engaged in the AGTO intervention through the AGTO participation index, which is the sum of six true/false items added to the mid and post coalition survey. Based on the Hall et al. model of categorizing the degree to which individuals *‘*use*’* an innovation [49-51], these items assess key markers of use including participation in training, reading the materials, planning, discussing the model with colleagues, securing resources, and receiving TA. Exposure to AGTO was also documented by TA providers recording hours of TA they delivered to each program, by AGTO step. The Participation Index and hours of TA have been shown to be related to prevention capacity and performance in a previous study of GTO [12,52]. A dichotomous measure was created if a user participated (AGTO participation index ≥1) at either mid or post. Most users who participated at mid also participated at post; however, there were a few cases in which respondents were documented to have used AGTO at mid but not at post (n = 4), or used at post, but not at mid (n = 19). Users at mid but not at post were assumed to hold residual knowledge or exposure at post. Preliminary analysis showed no significant differences between outcome measures of these groups and thus we felt confident characterizing participation as a single dichotomous measure of ever using (AGTO participation index ≥1) versus never using (AGTO participation index = 0).

### Program performance

A structured interview was used to assess the impact of AGTO on the implementation process, administered on the same timeline as the coalition survey. Prevention practitioners performance of tasks associated with high-quality prevention targeted by AGTO were captured through the interview. Whole programs are rated, not individuals, because programs operate as a unit. Using the interview responses, a set of ratings were made assessing performance of activities of seven key domains: goals and objectives, best practices, planning, process evaluation, outcome evaluation, continuous quality improvement, and sustainability. Because programs were mid-implementation the other three AGTO domains (needs assessment, fit, and capacity), all of which are pre-program implementation activities, were not assessed. The ratings come from 10 items (or *‘*components*’*) that assess how well each of the abovementioned activities are performed over the last year. Each component has seven response choices, described with specific behaviors, that range from *‘*highly faithful = 7*’* to *‘*highly divergent = 1*’* from ideal performance. This measure has been shown to be sensitive to change and reliable in previous GTO studies [12,53]. We calculated a score for each of the 10 components and a total score (i.e., average across all 10 components) for both the intervention and control-assigned programs at baseline and post. We then calculated the percent change for each component and the total score between baseline and post for all programs. In addition to the ratings, it was simply noted for each program whether they had conducted any process and outcome evaluation at each time point (yes/no).

Trained research staff conducted and audiotaped the structured interviews by telephone with the directors of 51 programs at baseline. The 32^d^ programs that were still operating two years later were interviewed again at post (AGTO = 17, control = 15). A second coder double-rated 10% of the programs using the audio recordings. The two raters discussed the scoring to reach a consensus when there were discrepancies. We calculated percent agreement and estimated a prevalence-adjusted bias-adjusted kappa (PABAK) [54] because of the potential impact on kappa from the relatively high prevalence of positive ratings. At baseline and post, respectively, the percent agreement between raters was 79% and 99% and the PABAK was 0.59 and 0.99.

### Perceptions of AGTO

After the intervention concluded, we conducted semi-structured interviews with staff from 13 programs (March to May, 2012) to gather practitioners’ perceptions of AGTO, focusing on ease of use and satisfaction with AGTO as well as the benefits and challenges they experienced with AGTO. These are all characteristics that influence implementation success and are consistent with the intervention characteristics identified by the CFIR. A convenience sample of staff that were consistently engaged in the project (i.e., no turnover) and significant users of AGTO were selected for these interviews because they were determined to be those most likely to have the knowledge and experience needed for the interview.

### Statistical analysis

#### Assessing changes in prevention capacity

We used two approaches to assess changes in capacity: an intent-to-treat analysis and because AGTO is a voluntary intervention, an analysis comparing users of AGTO to non-users. Our data was clustered by individuals within matched coalition, therefore, for the intent-to-treat analysis we fit a model with two sets of random effects, one for matched coalitions and one with random intercepts and linear functions of time to account for trajectories of repeated observations within respondent. The model included three fixed effect terms: group (AGTO versus control), time (baseline = 0 years, mid = 1 year, post = 2 years), and an interaction between group and time. The interaction term (i.e., *‘*difference of differences*’*) was tested to determine whether the slope of the best-fit line across all three time points varied by group. The data were analyzed in accordance with intervention assignment. For the AGTO user versus non-user analysis, we repeated the intent-to-treat models, but instead of comparing the AGTO to non-AGTO groups we compared AGTO users (AGTO participation index ≥1) to AGTO non-users (Index = 0). To ensure these groups were equivalent we conducted preliminary analyses to determine if there were significant differences in baseline capacity measures. Only one significant difference was detected in GTO behaviors (p = 0.047, SE = 3.629), and given that it was one of a large number of t-tests we do not anticipate this difference influenced the findings presented in this article.

### Assessing use of and exposure to AGTO

Means and percentages were calculated for the participation index, and its individual components, for both study groups.

### Program performance and its relationship to AGTO exposure

Given the small number of programs in this analysis, we were limited to primarily descriptive analyses. To assess the relationship between the exposure to AGTO (i.e., TA hours, by performance domain) and change in program performance, we aggregated across all AGTO programs and treated each AGTO domain as a case (i.e., n = 7), and calculated the relationship of TA hours spent on each domain with the amount of change exhibited in that domain’s Performance rating.

### Assessing perceptions of AGTO

We used constant comparative analysis to analyze structured interview data [55]. A member of the research team read each interview transcript and coded text to identify categories representing program staff perceptions of AGTO. Thirty-eight percent of transcripts were read and coded by two different team members. Inter-rater reliability was 0.86. After coding, the codes were sorted and categorized into themes. Thirty-one themes (e.g., It is easier to implement AGTO when it is integrated into routine activities) were identified and grouped into *categories* (e.g., factors that facilitated the implementation of AGTO).

## Results

### Prevention capacity of AGTO versus control

Model-based mean estimates at each of the time points are shown in Table 4 along with tests of the change over time within group and between groups. In the intent-to-treat analysis, assets efficacy within the control group significantly increased over time (a 2.6 point increase, SE = 1.23) between each time point, making a 5.2 increase from baseline to post; p = 0.035. The control group showed a decline in GTO behaviors (adjusted averaged decline of 4.3 points baseline to post) but the change was not significant (p = 0.055). The amount of change between the intervention and control groups did not significantly differ across the three time points for assets efficacy, GTO behaviors, or for any of the other prevention capacity scales in tests of condition by time interaction.

**Table 4:** **Intent to treat analysis: adjusted means (standard errors) on 0–100 capacity scales**^**i**^

	**Control**			**AGTO-treatment**							
**Outcome**	**Base**	**Mid**	**Post**	**Base**	**Mid**	**Post**	**Test**	**Estimate**	**df**	**t-statistic**	**p-value**
Assets efficacy	53.94 (2.78)	56.55 (1.9)	59.16 (1.59)	58.95 (2.85)	60.68 (1.96)	62.41 (1.6)	tx group	1.73	965	1.41	0.16
							non-tx group	2.61	965	2.12	0.03
							interaction	−0.88	965	−0.50	0.61
GTO efficacy	59.79 (2.64)	58.71 (1.85)	57.63 (1.64)	63.38 (2.7)	61.37 (1.91)	59.36 (1.65)	tx group	−2.01	967	−1.71	0.09
							non-tx group	−1.08	967	−0.92	0.36
							interaction	−0.93	967	−0.56	0.58
Assets behaviors	48.72 (3.06)	48.22 (2.31)	47.72 (2.1)	49.35 (3.12)	49.64 (2.37)	49.94 (2.12)	tx group	0.29	958	0.24	0.81
							non-tx group	−0.50	958	−0.40	0.69
							interaction	0.79	958	0.46	0.65
GTO behaviors	53.88 (2.48)	51.75 (1.69)	49.62 (1.4)	58.51 (2.53)	57.17 (1.75)	55.82 (1.44)	tx group	−1.35	960	−1.24	0.22
							non-tx group	−2.13	960	−1.92	0.05
							interaction	0.78	960	0.50	0.61
AGTO behaviors	38.44 (3.25)	38.47 (2.57)	38.5 (2.38)	40.92 (3.31)	42.25 (2.62)	43.59 (2.4)	tx group	1.34	958	1.10	0.27
							non-tx group	0.03	958	0.03	0.98
							interaction	1.31	958	0.75	0.45

^i^Models adjusted for repeated measures and clustered matched paired randomized design.

The clustering among the five outcomes was low. It was 0.00 for assets efficacy (95% CI, 00.0, 0.09) and GTO behaviors (95% CI, 00.0, 0.07), 0.01 for GTO efficacy (95% CI, 00.0, 0.09) and assets behaviors (95% CI, 00.0, 0.07), and 0.02 for AGTO behaviors (95% CI, 00.0, 0.07). All clusters were represented in the analyses.

### Use of and exposure to AGTO

The participation index showed that 47% of those in the AGTO group (N = 281) had used some portion of the intervention (participation index ≥1, M = 1.94, SD = 2.37) by reading AGTO materials (37%), participating in training (36%), talking to others about AGTO (33%) or making plans to use it (32%), receiving TA (30%), or securing resources for AGTO (24%). For the non-AGTO group, participation was at 20.6% (N = 64). Participation from reading most of the materials was 12.6%, 11.3% made plans to use, 7.7% talked to others, 7.1% participated in training, 5.5% secured or tried to secure resources, and 2.9% received TA. Mean use among the non-AGTO group was 0.48 (SD 1.09) and ranged from 1 to 5. Use of AGTO varied across each coalition (see Table 2).

#### Prevention capacity of users and non-users in the AGTO group

**Figure 2:**
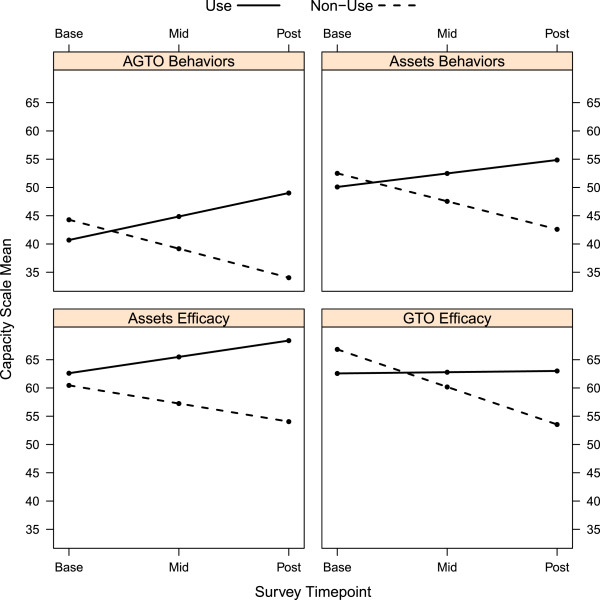
Plot of the any AGTO use vs. no AGTO use among participants assigned to the AGTO group: adjusted means on 0-100 capacity scales.

Four prevention capacity scales in the *‘*use/non-use*’* analyses (Table 5) had significant difference of difference tests: assets efficacy (p = 0.020), GTO efficacy (p = 0.005), assets behaviors (p = 0.003), and AGTO behaviors (p = 0.0003). The average difference across two time points between the AGTO use and AGTO non-use groups ranged from 6.1 to 9.3 points, resulting in a range of 12.2 to 18.6 from baseline to post. Figure 2 shows the model-based means at each time point for the four scales with significant difference of differences.

**Table 5:** **Any AGTO use versus no AGTO use among participants assigned to the AGTO group: adjusted means (standard errors) on 0–100 capacity scales**^**j**^

	**Non-use**			**Use**							
**Outcome**	**Base**	**Mid**	**Post**	**Base**	**Mid**	**Post**	**Test**	**Estimate**	**df**	**t-statistic**	**p-value**
Assets efficacy	60.46 (4.01)	57.25 (2.43)	54.05 (1.96)	62.61 (3.9)	65.49 (2.52)	68.38 (1.75)	user group	2.89	485	1.73	0.09
							non-user group	−3.21	485	−1.59	0.11
							interaction	6.09	485	2.33	0.02
GTO efficacy	66.83 (4.11)	60.18 (2.84)	53.54 (2.58)	62.57 (3.89)	62.79 (2.85)	63.01 (2.4)	user group	0.22	485	0.14	0.89
							non-user group	−6.65	485	−3.46	0.00
							interaction	6.86	485	2.79	0.01
Assets behaviors	52.5 (4.21)	47.55 (2.82)	42.6 (2.45)	50.09 (3.92)	52.48 (2.83)	54.86 (2.29)	user group	2.39	478	1.57	0.12
							non-user group	−4.95	478	−2.50	0.01
							interaction	7.34	478	2.94	0.00
GTO behaviors	59.01 (3.73)	54.56 (2.37)	50.12 (2.09)	61.24 (3.44)	60.82 (2.42)	60.4 (1.95)	user group	−0.42	478	−0.30	0.76
							non-user group	−4.45	478	−2.37	0.02
							interaction	4.02	478	1.72	0.09
AGTO behaviors	44.29 (4.05)	39.17 (2.58)	34.04 (2.25)	40.7 (3.85)	44.86 (2.67)	49.02 (2.09)	user group	4.16	478	2.67	0.01
							non-user group	−5.13	478	−2.55	0.01
							interaction	9.28	478	3.65	0.00

^j^Models adjusted for repeated measures and clustered matched paired randomized design.

### Program performance and its relationship to AGTO exposure

Noteworthy improvements for the AGTO programs were made in the domains of goals and process and outcome evaluation (Table 6). 64% of participating programs began a new process evaluation and 29% began a new outcome evaluation since receiving AGTO. Among the control group, only outcome evaluation had any improvement. The correlation between improvement in each domain and the TA hours spent on that domain was not statistically significant, possibly due to a small sample size [r(7) = 0.66, p = 0.102]. Best practices, sustainability, planning, and continuous quality improvement did not improve or declined, despite having some TA hours spent on them. However, with greater hours, more improvement was noted in goals and process and outcome evaluation.

**Table 6:** **Program performance ratings**^**k**^

	**AGTO (Overall TA hours, % Change correlation = 0.66) n = 17**							**Control n = 15**					
	**Scores at each time**			**% Change**			**TA hours spent baseline to post**	**Scores at each time**			**% Change**		
	**Baseline**	**Mid**	**Post**	**Baseline to Mid**	**Mid to post**	**Baseline to post**		**Baseline**	**Mid**	**Post**	**Baseline to mid**	**Mid to post**	**Baseline to post**
Goals	3.81	3.88	4.06	1.64	4.84	6.56	59.00	3.62	3.42	3.33	−5.5	−2.44	−7.8
Best practices	4.75	4.81	3.75	1.32	−22.08	−21.05	53.25	4.69	4.08	3.67	−12.98	−10.2	−21.86
Planning	4.94	4.38	4.25	−11.39	−2.86	−13.92	39.25	4.62	4.17	4	−9.72	−4	−13.33
Process evaluation	3.31	3.56	3.38	7.55	−5.26	1.89	65.75	3.88	3.75	3.58	−3.47	−4.44	−7.76
Outcome evaluation	2.78	3.03	3.00	8.99	−1.03	7.87	78.50	3.08	2.83	3.38	−7.92	19.12	9.69
CQI	3.69	3.06	3.28	−16.95	7.14	−11.02	36.50	3.81	2.14	2.67	−43.79	24.58	−29.97
Sustain	4.4	3.94	3.63	−10.51	−7.94	−17.61	56.00	3.92	2.15	3.58	−45.1	66.37	−8.66
TOTAL	3.95	3.81	3.62	−2.77	−3.88	−6.76	511.00	3.95	3.22	3.46	−18.35	12.71	−11.38

^**k**^Ratings lowest score = 1, highest = 7.

### Perceptions of AGTO

The interviews with staff who had used AGTO suggested some contextual factors that may both facilitate (e.g., key leader support) and hinder (e.g., staff turnover) the implementation of AGTO, as well as characteristics of the AGTO intervention that may have supported (e.g., access to a qualified TA provider) or challenged (e.g., complexity of the model) its implementation (Table 7). Factors that may facilitate implementation include whether AGTO was integrated into routine activities (31%), the number of people trained (23%), and key leader support (15%); whereas, barriers included diminishing funding (31%) and staff turnover (15%). Interviewees reported the AGTO process was beneficial because it offered access to a qualified TA provider (100%), a wide range of written support materials (92%), and peer-to-peer networking opportunities (85%), among other benefits. However, the process was also challenging for users who viewed it as cumbersome (54%), at times, complex (46%), and in competition with regular job duties (46%). Despite these challenges interviewees described a series of accomplishments as a result of engaging in AGTO, some of which sustained past the end of TA. Among the most notable accomplishments were that programs used more systematic processes for their work (100%), started collecting outcome evaluation data (100%), established stronger partnerships (92%), and integrated more evidence-based practices into their work (92%). Evaluation efforts were sustained by 77% of interviewees.

**Table 7:** **General categories and sub-categories of themes identified in structured interviews with AGTO programs (n = 13 participants)**^**l**^

**Category**	**Theme**	**Percent of interviews (n)**	**Illustrative quote**
Factors that facilitated AGTO implementation	When AGTO is integrated into routine activities	31% (4)	No illustrative quote^l^
	When more people are trained to use AGTO	23% (3)	[AGTO] spread across office of paid staff because the majority of staff participated in AGTO process.
	When key leaders support AGTO	15% (2)	No illustrative quote^l^
Barriers that constrained AGTO implementation	Diminishing funding climate	31% (4)	The funding levels for prevention just bottomed out in the middle of this project.
	Staff turnover due to funding	15% (2)	No illustrative quote^l^
Challenges with the AGTO process	Can be cumbersome to implement	54% (7)	No illustrative quote^l^
	A complex process	46% (6)	…It was kind of more towards the end really I kind of really grasped onto it. … this is a whole other field that I was not in.
	Competes for time with regular job duties	46% (6)	If it hadn’t been for [TA provider], I probably wouldn’t have [engaged with AGTO] – she was the one that keep me on task because I mean this was only just one part of my position.
	At times, misaligned with on-the-ground program operations	15% (2)	Sometimes it was very difficult to take a program in Maine and have it understood by people at a great distance. And sometimes the suggestions that we would get kind of showed that they didn’t quite get the program or an aspect of the program.
Benefits of the AGTO process	Access to a qualified TA provider	100% (13)	[It helped] …to have them be able to interpret….research-y language and translate that into, on the ground.. *‘*This is what they really mean, and this is what you can do with that.*’*
	Provided a wide range of written support materials	86% (12)	I think the manual is an absolute godsend, especially with the worksheets. It was really clear to understand. It was laid out in a way so that you could just flip to it and find something that might be helpful as far as embarking on a new project…
	Access to peer-to-peer networking opportunities	85% (11)	It was always interesting to talk to other people from other programs around the communities that were involved in AGTO. And you always got ideas from them…. They actually made me think more deeply about things and gave me a different perspective.
	Provided high-quality training	69% (9)	The training sessions that we’ve had have been extremely beneficial, and I’ve had nothing but positive feedback from our coalition members when we’ve had them.
	Proactive approach	54% (7)	We would have specific things that we were doing. It was always pretty specific. There wasn’t too much general, though after our specific goals we be met I would just talk to her about just some general problems areas.
	Emphasized/prioritized collection and analysis of data	54% (7)	I mean, adding outcome, legitimate outcome data collection and looking at behavioral change was a significant difference because of AGTO.
	Access to larger body of experts via the Project Leadership Team	31% (4)	I really think that [the principal investigator] and the whole team involved in providing the support and TA have been superb. We’re very blessed to have that level and quality of support because it’s certainly made a difference here.
	Worked for participants regardless of baseline capacity	31% (4)	No illustrative quote^l^
Program/coalition accomplishments	Learned to be more systematic and intentional about work	100% (13)	[The AGTO] process, because it’s rigorous and logical and exacting, makes you more realistic in your thinking about your situation …. And what you can do to affect [it] and what you can’t do.
	Started collecting outcome evaluation data	100% (13)	The evaluation tools and how to use data in a way that informed decision making, I think, was probably the biggest takeaway from this experience.
	Paved the way for stronger relationships and partnerships	92% (12)	I think we better prepared ourselves even for approaching [partners] because we were able to make our programs much more defined.
	Integrated more evidence-based practices into their work	92% (12)	We’re working towards having everything be evidence-based.
	Took inventory of needs and resources to better focus them	77% (10)	[We learned]…what we have available in terms of partner organizations, their resources, the coalition’s resources, and … how can we focus those so [they]…produce change.
	Improved youth voice in program/coalition activities	62% (8)	Youth are involved in program planning, implementation, and evaluation.
	Improved communication about program/coalition	38% (5)	No illustrative quote^l^
Aspects of AGTO sustained after the project ended	Continued to evaluate their program/coalition	77% (10)	We’ve had the physical presence of people who are keeping [evaluation efforts] going.
	Continued to work all 10 steps of AGTO	38% (5)	AGTO becomes endemic to the work. Even though TA ends you don’t unlearn the skills and knowledge developed.
	Generalized AGTO to other activities/topic areas	38% (5)	No illustrative quote^l^
	Continued to use manuals and other written support materials	38% (5)	Just a couple of weeks ago …I was getting the Getting To Outcomes with Developmental Assets [manual] out. I was looking through it and I see things that I haven’t seen before.
	Continued to use logic model to guide program/coalition work	23% (3)	No illustrative quote^l^
	Not able to sustain AGTO	23% (3)	I think that support [TA] was crucial and, without it, it really hasn’t--it’s been hard to sustain.

^*l*^*Cells without illustrative quotes were left blank because there was no single quote that illustrated the theme, but was the result of a much longer narrative that includes exchanges between the interviewer and interviewee.*

## Discussion

Using the implementation research typology outlined in CFIR, this study evaluated the AGTO intervention’s impact on the capacity of individual prevention practitioners (i.e., CFIR’s individual characteristics) and the performance of whole programs (CFIR’s implementation process), while accounting for several factors in CFIR’s inner setting domain. Contrary to study hypothesis one, the intent-to treat analyses showed prevention practitioners in the control group demonstrated significant gains in assets efficacy, although the control group’s GTO behaviors declined. There were no other significant changes over time for either group on any of the other measures in these analyses. The variation in AGTO use within the treatment group and use of AGTO in the control group potentially contributed to these non-significant findings. There are a few possible explanations for why individuals in the non-AGTO group reported use of AGTO. Since AGTO is a set of well-known practices for the implementation of high-quality prevention, it is possible that individuals would have been exposed to or using some of these practices. In addition, the AGTO book is freely available for purchase by anyone and in some instances control coalition members attended AGTO trainings.

While intent-to-treat analyses are more traditional, they may be overly conservative given that GTO is a voluntary intervention. To help address these potential analytic limitations, the *‘*use/no-use*’* analyses were conducted and showed, consistent with study hypothesis two, significant differences between those with greater exposure to and use of AGTO. Another advantage of these analyses is that they avoid the potential biases of contamination (i.e., control groups using AGTO) and differential response rates between intervention and control groups common in implementation research. We also found that whole prevention programs in the AGTO group demonstrated significant gains in certain domains of prevention performance (i.e., performing various prevention tasks with high quality) overall and related to their use of TA (hypothesis three).

In the interviews, practitioners stated that they generally valued AGTO, especially the TA component, and found it helpful to their work. However, practitioners also experienced challenges of not having enough time and resources to sufficiently carry out AGTO, further highlighting the need for TA. Prior research has indicated that these types of costs (i.e., time and resources) can be negatively associated with implementation [56]. The importance and contribution of TA to improving the implementation process is consistent with past GTO [57] and other studies that involve TA [58,59].

Although replicating earlier findings on GTO’s impact on capacity [12,52] and program performance [12,52,53], this study is the first of its kind to show that an implementation support intervention-AGTO-yielded improvements in practitioner capacity and consequently in program performance on a larger sample of practitioners and programs using a randomized controlled design. These findings support the theory behind implementation support interventions generally and AGTO specifically, which posit that improving practitioner capacity to engage in the activities of the 10 steps of AGTO will consequently improve program performance. The final link in the GTO theoretical chain (implementation support→capacity→program performance→youth outcomes) is currently being tested in an ongoing multi-site, randomized trial (1R01HD069427-01, Chinman, PI), with results expected in 2014.

Findings from the current study show that using AGTO helps improve the quality of real world implementation. There are other interventions that aim to build capacity-e.g., in the area of alcohol and drug prevention (e.g., Communities That Care [60] and PROmoting School-community-university Partnerships to Enhance Resilience [61]). However, unlike these interventions that provide support and resources to help community practitioners select and begin implementing evidence-based prevention programs, AGTO engages existing programs (whether evidence-based or home-grown) and the leadership of organizations that house these programs to improve their quality through support with no additional financial resources for implementation. As such, the goal of the AGTO intervention is to help the leadership of community organizations to integrate the practices AGTO targets into routine operations.

The methods used in the current study also have implications for broader implementation research using the CFIR. The study used measures to assess the quality of the implementation process as well as the relevant capacity of practitioners (i.e., individuals involved) that could be adapted to many other interventions, and exist already for interventions in the areas of substance abuse prevention, positive youth development, prevention of teen pregnancy and sexually transmitted infections, and homelessness prevention. These measures were used in an randomized controlled trial and our analytic approach addressed certain challenges of conducting research on real-world implementation (e.g., the turnover of membership at participating coalitions, differential response rates between intervention and control groups). More innovative methods and more acceptance of nontraditional research methods will be needed to capture the complex, multi-level and transient status of constructs in the real-world as the study of implementation evolves.

### Limitations

The interviews to gather perceptions of AGTO were done with a convenience sample, which could have introduced bias into those findings. This was in part a result of diminishing budgets for prevention in Maine, which caused a sizable dropout of programs between baseline and post (although equivalent across groups). A sample of 32 programs with complete program performance ratings is modest. Therefore the percent change analyses should be interpreted with caution. Future studies that recruited even larger numbers of schools, communities, or coalitions would provide the opportunity to use inferential statistics to better test the relationships between TA and various prevention tasks. Further, TA hours, even by domain, is not the only important measure of TA; quality of the TA, timing about when it was given (e.g., planning before evaluation), and type of program are other factors to be explored in future research [62,63]. Also, because the use versus non-use groups were not randomly assigned, there may have been selection biases within use versus non-use analyses-e.g., practitioners who engaged in the intervention may have been more likely to improve than those who did not. However, this bias may be mitigated somewhat because the capacity scores with significant group (user versus non-user) X time (pre-mid-post) interactions were not significantly different at baseline among the use versus non-use groups. It is important to note, capacity-building interventions like AGTO are voluntary and thus this analysis represents a real-world test.

## Conclusions

The types of programs and practitioners AGTO targeted in this study are typical of prevention efforts in many communities. Despite challenges, AGTO helped programs that made use of it make critical improvements in the quality of their work. Implementation support interventions like AGTO can help ensure investments in real world prevention programming are conducted with high quality and consequently yield positive outcomes.

## Endnotes

^a^Getting To Outcomes and GTO are trademarks registered by University of South Carolina and RAND.

^b^US Census 2000.

^c^According to the 2006 Maine Youth Drug and Alcohol Use Survey (http://www.maine.gov/dhhs/osa/data/mydaus/mydaus2006.htm).

^d^Nine programs ended between the time the study was planned and begun, leaving only 51 programs at baseline. 19 more programs ended between baseline and post.

## Abbreviations

AGTO: Assets getting to outcomes; CFIR: Comprehensive framework for implementation research; GTO: Getting to outcomes; PABAK: Prevalence-adjusted bias-adjusted kappa; TA: Technical assistance.

## Competing interests

The authors declare that they have no competing interests.

## Authors’ contributions

AP carried out the qualitative interviews, qualitative analysis of interviews and drafted the manuscript. MS, SP and PM designed and performed the statistical analyses. PS participated in the drafting of the manuscript. JA, MC, and PE conceived of the study, and participated in its design and coordination and helped to draft the manuscript. All authors read and approved the final manuscript.

## Authors’ information

Joie Acosta is a Behavioral Scientist at the RAND Corporation and specializes in research and evaluation to improve the implementation of evidence-based positive youth development, substance abuse and mental health prevention and treatment programs. From this work, she co-authored reports on building capacity for outcomes measurement among human service organizations and improving the implementation of social services and behavioral health care.

Matthew Chinman is a Senior Behavioral Scientist at the RAND Corporation, whose recent focus has been to develop strategies to enhance the capacity of community-based practitioners. He co-developed Getting To Outcomes® (GTO), a model designed to help community organizations to better plan, implement, and self-evaluate programs across a number of domains. He has led the successful testing of the several Getting To Outcomes-based guides in the areas of substance abuse prevention, underage drinking prevention, and youth development.

Patricia Ebener is a senior researcher with RAND's Drug Policy Research Center and a senior survey director with RAND's Survey Research Group. She has collaborated on community based participatory research and quality of prevention and treatment services projects for over twenty years.

Patrick Malone, a quantitative and social psychologist, is an Associate Professor of Psychology at the University of South Carolina. He specializes in methods for latent variable modeling and longitudinal analysis for adolescent health risk behaviors.

Susan Paddock is a Senior Statistician at the RAND Corporation. Dr. Paddock’s research interests include statistical methods development and statistical applications to substance abuse treatment studies.

Andrea Phillips is a Project Associate at the RAND Corporation.

Peter C. Scales is Senior Fellow at Search Institute. For nearly four decades, he has been widely recognized nationally, and more recently, internationally, for his research, direct service work, and comprehensive writings on promoting positive child and youth development. He is the world's leading researcher on the Developmental Assets approach to positive development, has led more than 15 large-scale or nationally representative studies, and international studies in a dozen countries on five continents, and authored or co-authored more than 250 publications, including 10 books, and 75 peer-reviewed journal articles and book chapters.

Mary Ellen Slaughter is a statistical/quantitative analyst at RAND with statistical expertise in regression analysis, categorical data analysis, survival analysis, and multivariate analysis. She also has experience with cost-effectiveness analysis, large health care databases, geographic information systems, and is proficient in SAS and R statistical programming.

## Supplementary Material

Additional file 1: Table S1 CONSORT Checklist (Version 2010). Click here for file
